# Effects of Ethanolic Lyophilized and Nanoparticle Extracts of Cilo Wormwood (*Artemisia haussknechtii* Boiss.) Leaves on Histopathological Findings Antioxidant Defence System and Serum Biomarker Constituents in Diabetic Rats

**DOI:** 10.1007/s12010-026-05751-5

**Published:** 2026-06-04

**Authors:** Musa İşnas, İsmail Çelik, Bedia Bati̇, Nevra Aydemi̇r Celep

**Affiliations:** 1 Department of Molecular Biology and Genetics, Sciences Faculty, Van Yuzuncu Yil University, Van, Turkey; 2 Faculty of Education, Department of Mathematics and Science Education, Van YuzuncuYil University, Van Yuzuncu Yil University, Van, Turkey; 3 Department of Histology and Embryology, Faculty of Veterinary Medicine, Atatürk University, Erzurum, Turkey

**Keywords:** *Artemisia haussknechtii*, Antioxidant enzymes, Oxidative stress, Liver, Kidney, Pancreas

## Abstract

Molecular and structural abnormalities triggered by oxidative stress are key determinants of diabetes pathophysiology, and research into natural therapeutic agents targeting these mechanisms is gaining speed. This study aimed to evaluate the effects of *Artemisia haussknechtii* leaves ethanolic lyophilized and nanoparticle extract forms on glycemic regulation, oxidative stress markers, and tissue integrity in an experimental diabetes model. A total of 48 female rats were divided into six groups; diabetes was induced with 45 mg/kg STZ (i.p.). DYEE and DYNE were administered orally at doses of 50 and 100 mg/kg for 21 days. Weekly fasting glucose and body weight were monitored. At the end of the study, serum biochemistry, erythrocyte and tissue oxidative stress markers (MDA, GSH, CAT, GPx, SOD, GR, TAS, TOS) were analyzed; liver, kidney, and pancreas tissues were evaluated histopathologically. Diabetic rats exhibited persistent hyperglycemia, body weight loss, dyslipidemia, elevated liver and kidney injury biomarkers, increased lipid peroxidation (MDA), and total oxidant status (TOS), accompanied by marked suppression of antioxidant defense systems. Treatment with *Artemisia haussknechtii* extracts significantly ameliorated these alterations in a dose-dependent manner. Notably, the high-dose nanoparticle formulation (DYNE2) produced the most pronounced improvements, reflected by reduced blood glucose levels, improved lipid profile, normalization of insulin, HbA1c and c-peptide levels, decreased MDA and TOS, and restoration of antioxidant parameters toward control values. Histopathological findings corroborated the biochemical data, demonstrating substantial attenuation of diabetes-induced tissue damage, particularly in the DYNE2 group. *Artemisia haussknechtii* extract, particularly in its nanoparticle form, has demonstrated antihyperglycemic, antioxidant, and tissue-protective effects in experimental diabetes. The findings suggest that the plant is a promising candidate for complementary treatment against diabetes-related oxidative stress and organ damage.

## Introduction

Diabetes mellitus (DM) is a chronic metabolic disorder characterized by impaired carbohydrate, lipid, and protein metabolism, resulting from insufficient insulin secretion or impaired insulin action [1, 2]. This hyperglycemia-based condition is associated with the development of serious complications such as cardiovascular diseases, nephropathy, retinopathy, and hepatic dysfunction [3, 4]. Prolonged hyperglycemia leads to oxidative stress by causing excessive production of reactive oxygen species (ROS) and weakening antioxidant defense systems. This process is considered a fundamental mechanism in the pathophysiology of diabetes and the progression of its complications [5, 6].

Oxidative stress develops as a result of the disruption of the balance between free radicals and antioxidant defenses. In diabetic individuals, it weakens β-cell function and increases insulin resistance by enhancing lipid peroxidation, protein oxidation, and DNA damage [7–9]. Strategies to reduce oxidative stress are critically important for preventing diabetic complications [10, 11]. In this context, the antioxidant defense system consists of enzymatic components such as SOD, CAT, GSH-Px, and GR; and non-enzymatic molecules such as GSH, vitamin C, and vitamin E. A significant loss of activity in this system is observed in diabetes. Parallel to this, it has been reported that oxidative stress markers such as MDA increase and the TAS/TOS balance is disrupted [12–16]. Among these, MDA serves as a key indicator of lipid peroxidation and membrane damage; GSH represents the primary non-enzymatic antioxidant responsible for scavenging reactive oxygen species; SOD and CAT are frontline enzymatic defenses that neutralize superoxide radicals and hydrogen peroxide, respectively; GPx and GR work in concert to maintain glutathione homeostasis; while TAS and TOS provide a comprehensive assessment of the overall oxidant-antioxidant balance in biological systems.

The progression of diabetes leads to both morphological and functional impairments in metabolically critical organs such as the liver, kidneys, and pancreas [17, 18]. Glucose regulatory mechanisms are impaired in the liver, while filtration functions and electrolyte balance are disrupted in the kidneys. Oxidative stress associated with long-term hyperglycemia causes inflammation, necrosis, and cellular damage in these organs [19, 20]. In the pancreas, β-cell damage leads to decreased insulin production and further disruption of glycemic balance [21, 22].

In recent years, plant extracts rich in phenolic and flavonoid compounds have been shown to have significant potential in reducing diabetes-related oxidative stress and strengthening the antioxidant defense system [8, 9, 23, 24]. Artemisia species belonging to the Asteraceae family stand out in this context due to their anti-inflammatory, antihyperglycemic, and antioxidant activities [25, 26]. Artemisia species have a wide range of uses as flavorings in food products and are considered in traditional medicine as antispasmodic, antiparasitic, antibacterial, and antirheumatic agents; they are also reported to be used in the treatment of malaria, hepatitis, infections, inflammatory conditions, and menstrual cramps [27–30]. This pharmacological diversity is based on a broad phytochemical profile, including terpenoids, phenols, flavonoids, coumarins, sesquiterpene lactones, lignans, and alkaloids [31].

The streptozotocin (STZ)-induced rat model is one of the most widely used and well-validated experimental models of type 1 diabetes mellitus, as STZ selectively destroys pancreatic β-cells through alkylation of DNA, thereby mimicking the insulin-deficient hyperglycemic state observed in clinical diabetes. Various studies have demonstrated that Artemisia species reduce glucose levels, improve lipid metabolism, and exert regulatory effects on oxidative stress parameters in streptozotocin (STZ)-induced diabetic models [32–35]. In particular, *A. judaica* has been reported to lower glucose levels by exhibiting insulin-like effects [36]. *Artemisia haussknechtii* Boiss. (Cilo yavşanı), which grows naturally in the Eastern Anatolia Region of Turkey, is a species frequently used in folk medicine for the treatment of hyperglycemia and metabolic disorders [37, 38]. This species has been reported to exhibit antibacterial and metal chelating activity [39] and to contain secondary metabolites with antioxidant properties, such as camphor, 1,8-cineole, borneol, and linalool [40, 41]. Additionally, its cytotoxic, antifungal, and insecticidal effects are documented in the literature [42, 43].

The development of nanoparticle-based systems to enhance the bioavailability, stability, and delivery of plant extracts to target tissues has become an important area of research in recent years. Solid lipid nanoparticles (SLNs) enhance the biological activity of plant compounds by enabling their controlled release and offer advantages, particularly in reducing oxidative stress [44, 45]. Recent studies have also demonstrated that nanoformulations of Artemisia species enhance antioxidant and antihyperglycemic effects [46–48].

To the best of our knowledge, this is the first study to simultaneously evaluate the antihyperglycemic, antioxidant, hypolipidemic, and tissue-protective effects of both lyophilized ethanolic extract and solid lipid nanoparticle formulation of Artemisia haussknechtii Boiss. in an STZ-induced experimental diabetes model. The novelty of this work lies in three key aspects: (i) the pharmacological characterization of an understudied endemic species from Eastern Anatolia, (ii) the direct comparison of conventional and nanoparticle-based delivery systems at two dose levels, and (iii) the comprehensive multi-organ histopathological and biochemical assessment providing an integrative evaluation of diabetes-induced systemic damage and its attenuation. Therefore, this study aimed to evaluate the effects of ethanolic lyophilized and solid lipid nanoparticle extracts of *Artemisia haussknechtii* Boiss. on glycemic regulation, lipid profile, serum biochemical markers, oxidative stress parameters (MDA, GSH, SOD, CAT, GPx, GR, TAS, TOS), and histopathological alterations in the liver, kidney, and pancreas of streptozotocin-induced diabetic rats.

## Materials and Methods

**Fig. 1:**
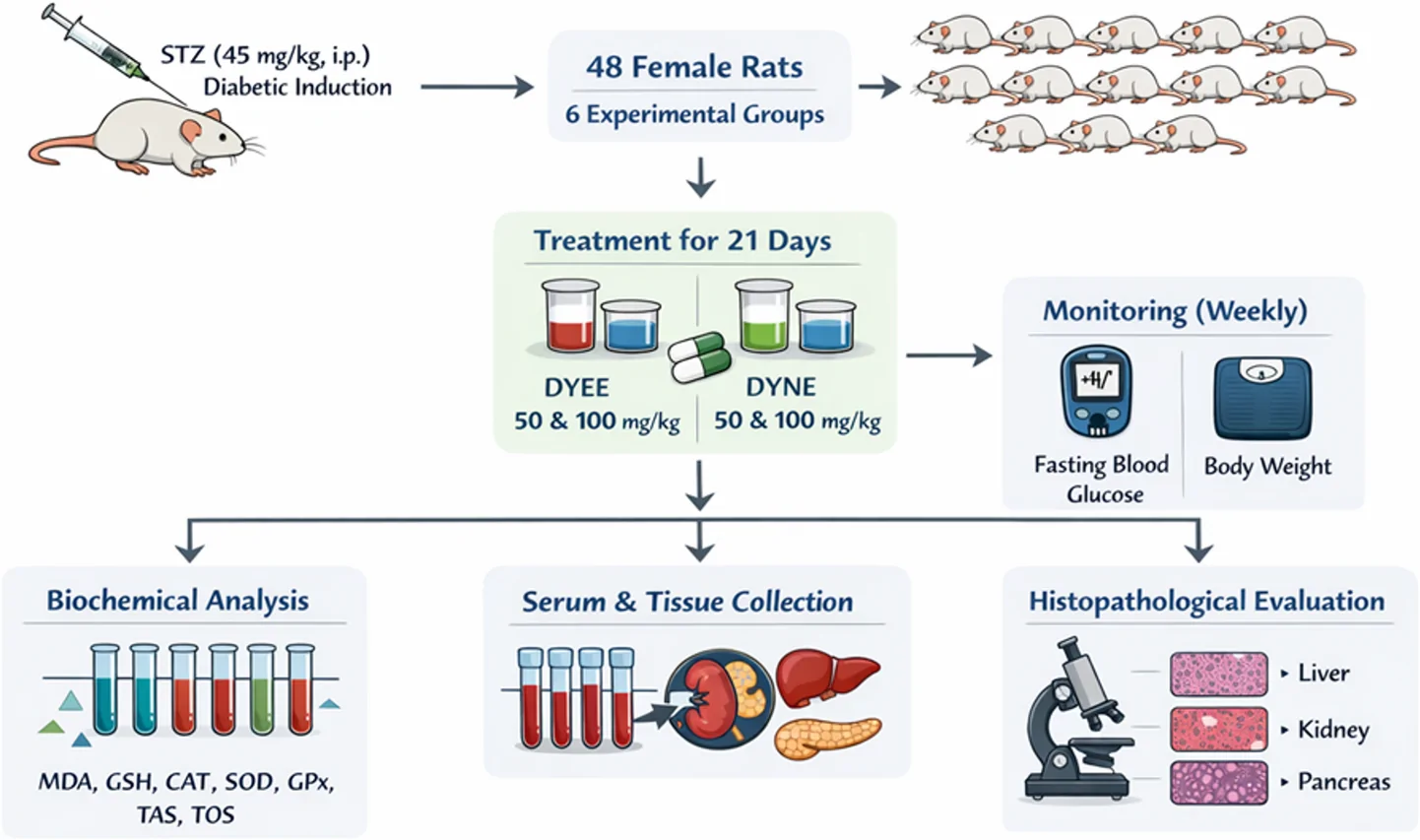
Schematic illustration of the experimental design used to evaluate the effects of *Artemisia haussknechtii* leaf ethanol extract (DYEE) and its nanoparticle formulation (DYNE) on the antioxidant system and histopathological alterations in streptozotocin-induced diabetic rats. Diabetes was induced by a single intraperitoneal injection of streptozotocin (STZ; 45 mg/kg) in 48 female rats, which were subsequently divided into six experimental groups. DYEE and DYNE were administered orally at doses of 50 and 100 mg/kg for 21 consecutive days. Fasting blood glucose levels and body weight were recorded weekly throughout the experimental period. At the end of the study, serum, erythrocyte, and tissue samples were collected for the assessment of oxidative stress and antioxidant parameters (MDA, GSH, CAT, SOD, GPx, TAS, and TOS). In addition, liver, kidney, and pancreas tissues were subjected to histopathological examination

The overall experimental design and treatment protocol employed in this study are summarized schematically in Fig. 1.

### Chemicals

Streptozotocin (STZ; 2-deoxy-2-(3-methyl-3-nitrosoureido)-D-glucopyranose), ketamine, superoxide dismutase (SOD), and glutathione peroxidase (GSH-Px) analysis kits, total antioxidant capacity (TAS) and total oxidant level (TOS) kits were used in the study. In addition, hydrogen peroxide (H₂O₂), potassium dihydrogen phosphate (KH₂PO₄), disodium hydrogen phosphate (Na₂HPO₄), dithiobis-(2-nitrobenzoic) acid (DTNB), sodium citrate, ethylenediaminetetraacetic acid (EDTA), Tris(hydroxymethyl)aminomethane hydrochloride (Tris-HCl), hydrochloric acid (HCl), β-nicotinamide adenine dinucleotide phosphate (NADPH), reduced glutathione (GSH), oxidized glutathione (GSSG), 1-chloro-2,4-dinitrobenzene (CDNB), ethanol, sodium chloride (NaCl), sodium hydrogen phosphate (NaHPO₄), Trolox, sodium dihydrogen phosphate (NaH₂PO₄), butylated hydroxytoluene (BHT), thiobarbituric acid (TBA), trichloroacetic acid (TCA), sodium hydroxide (NaOH), and metaphosphoric acid were used in analytical procedures. Hematoxylin, sodium thiosulfate, acid fuchsin, phosphotungstic acid, and aniline blue stains were obtained for histological evaluations. All chemicals were of analytical grade purity and were obtained from reliable commercial sources.

### Experimental Animals

All experimental procedures were conducted in accordance with the guidelines for the care and use of laboratory animals and were approved by the Van Yüzüncü Yıl University Local Animal Experimentation Ethics Committee (Approval date: January 27, 2022; Approval number: 2022/01–13). The study used 3-4-month-old female Wistar albino rats weighing 150–250 g. Female rats were preferred in this study as they have been widely used in STZ-induced experimental diabetes models in the literature and have been shown to exhibit consistent glycemic responses following STZ administration [49, 62]. Furthermore, the use of a single sex eliminates potential confounding effects of sex-related hormonal variability on oxidative stress parameters and metabolic outcomes, thereby increasing the internal validity of the experimental findings. The animals were obtained from the Experimental Medicine Application and Research Center of Van Yüzüncü Yıl University. During the experiment, the rats were kept at a temperature of 25 ± 1 °C, in a 12-hour light/12-hour dark cycle; they had ad libitum access to standard pellet feed and water.

### Experimental Design

Throughout the study, the ethanolic lyophilized extract of *Artemisia haussknechtii* is referred to as DYEE (Diyabetik Yavşan Etanolik Ekstrakt) and the solid lipid nanoparticle-loaded ethanolic extract is referred to as DYNE (Diyabetik Yavşan Nanopartikül Ekstrakt). The numerical suffixes 1 and 2 denote the low dose (50 mg/kg) and high dose (100 mg/kg) treatment groups, respectively. A total of 48 rats were randomly assigned into six groups (*n* = 8):

- Normal Control (NC): Non-diabetic rats with no treatment.
- Diabetic Control (DC): Diabetes was induced using streptozotocin (STZ) at 45 mg/kg bw dosage.
- DYEE1: Diabetic rats treated with *Artemisia haussknechtii* Boiss. leaves ethanolic lyophilized extract (50 mg/kg, orally via gavage).
- DYNE1: Diabetic rats treated with *Artemisia haussknechtii* leaves ethanolic nanoparticle extract (50 mg/kg. orally via gavage).
- DYEE2: Diabetic rats treated with *Artemisia haussknechtii* leaves ethanolic lyophilized extract (100 mg/kg. bw orally).
- DYNE2: Diabetic rats treated with *Artemisia haussknechtii* leaves ethanolic nanoparticle extract (100 mg/kg, bw. orally via gavage).

Diabetes was induced by a single intraperitoneal (i.p.) injection of STZ at a dose of 45 mg/kg, dissolved in 0.1 M citrate buffer (pH 4.5). This dose was selected based on well-established experimental protocols in the literature, as 40–50 mg/kg STZ administered intraperitoneally to rats has been consistently demonstrated to induce stable type 1-like diabetes mellitus by selectively destroying pancreatic β-cells, while minimizing non-specific systemic toxicity [49, 61, 62]. Seventy-two hours after administration, rats with blood glucose levels above 200 mg/dL, measured from tail vein blood samples, were considered diabetic [49]. Extract treatments were administered orally by gavage for 21 days, and body weights and blood glucose levels were monitored weekly.

### Plant Material and Lyophilized Extract Preparation

*Artemisia haussknechtii* Boiss. plant was collected from the rural area of Taşbaşı Village, Hakkâri Province, identified by botanical experts, and recorded in the VANF Herbarium under number 16,284. The plant leaves were dried in the shade and extracted with 80% ethanol according to the modified Dalar and Konczak [50] method. After homogenization and filtration, the liquid extract obtained was evaporated in a rotary evaporator and dried in a lyophilizer to produce a powder. The extracts were stored at − 20 °C until analysis.

### Nanoparticle Extract Preparation

The solid lipid nanoparticle (SLN) form of the plant extract was prepared based on the microemulsion–sonication method described by Ayan et al. [51]. For this purpose, Compritol (glyceryl behenate) was used in the lipid phase, while soy lecithin was preferred as the lipophilic surfactant and Tween 80 as the hydrophilic surfactant. The lyophilized plant extract was added to the lipid phase, and the mixture was homogenized by ultrasonication. The resulting hot microemulsion was slowly dripped into cold pure water to form SLNs. The obtained nanoparticles were lyophilized to convert them into powder form and stored at + 4 °C. Based on previously published stability data for similar SLN formulations, storage at + 4 °C has been demonstrated to preserve physicochemical properties including particle size, zeta potential, and encapsulation integrity for a period of up to 3 months, while storage at 25 °C may lead to particle aggregation and gradual degradation of bioactive components over time [85]. Stability characterization of the current formulation under defined storage conditions is planned as part of future work. The prepared *Artemisia haussknechtii*-loaded SLNs were characterized in terms of various physicochemical parameters such as stability, particle size, zeta potential, and encapsulation efficiency.

### Collection of Blood and Tissue Samples

On day 21 of the experiment, rats were anesthetized with 10% ketamine and blood samples were collected from the heart. Blood in EDTA tubes was used for erythrocyte analysis, while blood in biochemistry tubes was used for serum biochemistry tests. Liver, kidney, and pancreas tissues were removed, and a portion was stored in 10% formalin for histopathological examination, while the remaining portions were stored at − 80 °C for oxidative stress analyses [52].

### Serum Biomarker Constituents Assay

Serum biochemical parameters including insulin (pg/mL), HbA1c (%), C-peptide (pg/mL), alanine aminotransferase (ALT, U/L), aspartate aminotransferase (AST, U/L), urea (mg/dL), creatinine (CRE, mg/dL), lactate dehydrogenase (LDH, U/L), high-density lipoprotein cholesterol (HDL, mg/dL), low-density lipoprotein cholesterol (LDL, mg/dL), triglycerides (TG, mg/dL), and total cholesterol (TC, mg/dL) were measured using a fully automated biochemical analyzer (COBAS 8000, Roche Diagnostics, Germany; Serial No. 1296-08) with commercially available assay kits, according to the manufacturer’s instructions.

### Determination of Oxidative Stress and Antioxidant Parameters

The following analyses were performed in erythrocyte, liver, and kidney tissue supernatants: Malondialdehyde (MDA) levels were determined spectrophotometrically at 532 nm using the thiobarbituric acid reactive substances (TBARS) method as described by Slater [53] and Jain et al. [54], and were used as an indicator of lipid peroxidation and oxidative membrane damage. Reduced glutathione (GSH) levels were measured at 412 nm using the DTNB (5,5′-dithiobis-2-nitrobenzoic acid) colorimetric method according to Beutler et al. [55] and Rizzi et al. [56]. Glutathione reductase (GR) activity was assessed spectrophotometrically at 340 nm based on the rate of NADPH oxidation as described by Carlberg and Mannervik [57]. Catalase (CAT) activity was determined by monitoring the rate of hydrogen peroxide (H₂O₂) decomposition at 240 nm following the method of Aebi [58]. Glutathione S-transferase (GST) activity was measured at 340 nm using 1-chloro-2,4-dinitrobenzene (CDNB) as the substrate according to Mannervik and Guthenberg [59]. Superoxide dismutase (SOD) and glutathione peroxidase (GPx) activities were determined using commercially available enzyme-linked immunosorbent assay (ELISA) kits according to the manufacturer’s instructions. Briefly, erythrocyte and tissue homogenate samples were added to microplate wells pre-coated with specific antibodies, incubated at 37 °C, and absorbance values were measured at 450 nm using a microplate reader following the addition of substrate and stop solutions. Total antioxidant status (TAS) and total oxidant status (TOS) were determined using commercially available micro-ELISA kits based on automated colorimetric methods, and results were expressed in mmol Trolox equivalent/L and µmol H₂O₂ equivalent/L, respectively.

### Histopathological Examinations

Liver, kidney, and pancreas tissues collected at the end of the experimental period were fixed in 10% neutral buffered formalin for 48 h. Following fixation, tissues were dehydrated through a graded ethanol series (70%, 80%, 90%, 96%, and 100%), cleared in xylene, and embedded in paraffin blocks. Serial sections of 5 μm thickness were obtained using a rotary microtome. For routine histological evaluation, sections were stained with hematoxylin and eosin (H&E). In addition, Masson’s trichrome staining was applied to assess collagen deposition and connective tissue alterations. Stained sections were examined under a Zeiss AXIO Scope.A1 light microscope at ×20 magnification, and digital images were captured using the integrated camera system. Histopathological findings including cellular degeneration, vacuolization, necrosis, inflammatory cell infiltration, sinusoidal dilatation, tubular damage, glomerular atrophy, and islet cell loss were evaluated independently by a veterinary histologist blinded to the experimental groups. Severity of each finding was graded semi-quantitatively using a numerical scoring system: 0 = absent, 1 = mild, 2 = moderate, 3 = severe, 4 = very severe.

### Statistical Analysis

Data are expressed as mean ± standard deviation (X ± SD). Differences between groups were evaluated using one-way analysis of variance (One-Way ANOVA) and Tukey post hoc test; *p* < 0.05 was considered statistically significant [60].

## Results

### Physicochemical Characterization of Nanoparticles

**Fig. 2:**
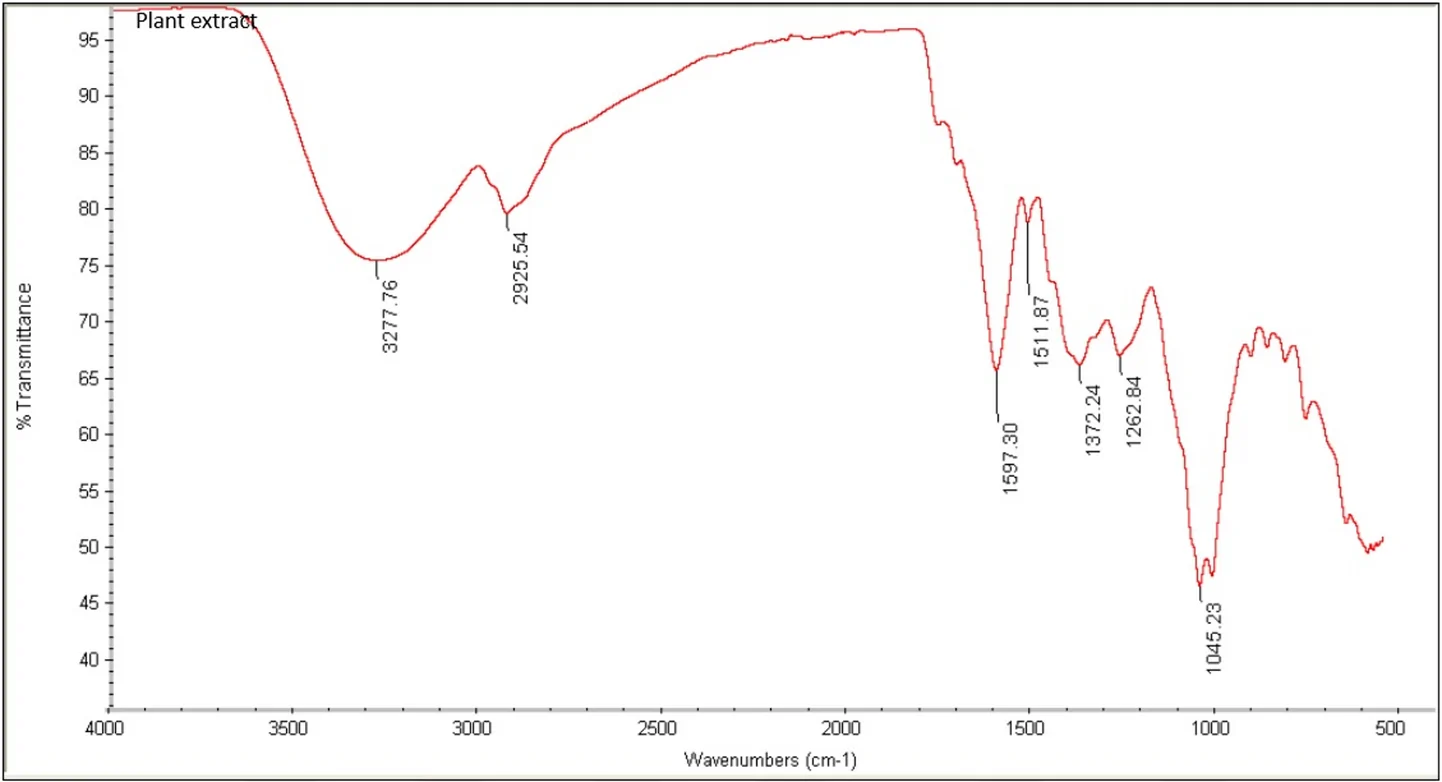
FT-IR spectrum of lyophilized *Artemisia haussknechtii* leaf ethanolic extract. Characteristic absorption bands are observed at 3277.76 cm⁻¹ (O-H stretching), 2921.54 cm⁻¹ (C-H stretching), 1811.87 cm⁻¹ (C = O stretching), 1597.30 cm⁻¹ (C = C aromatic stretching), 1372.24 and 1292.94 cm⁻¹ (C-H and C-O bending/stretching), and 1045.23 cm⁻¹ (C-O-C stretching)

**Fig. 3:**
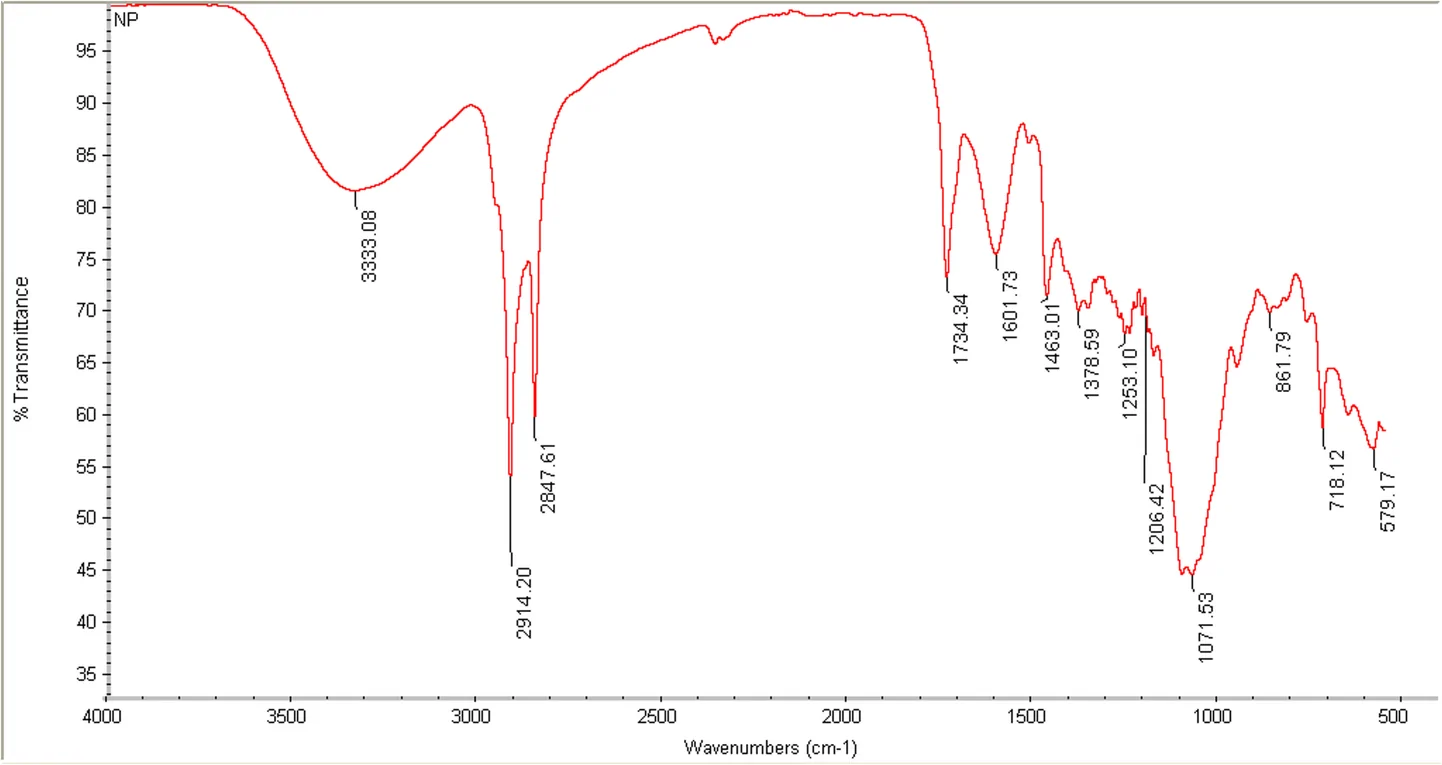
FT-IR spectrum of solid lipid nanoparticle-loaded *Artemisia haussknechtii* ethanolic extract. Key absorption bands are identified at 3303.08 cm⁻¹ (O-H stretching), 2947.61 and 2914.20 cm⁻¹ (C-H stretching), 1734.34 cm⁻¹ (C = O ester stretching of Compritol lipid matrix), 1601.79 cm⁻¹ (C = C aromatic stretching), 1206.42 and 1071.53 cm⁻¹ (C-O-C stretching). Peak shifts relative to the free extract spectrum confirm physicochemical interaction between the bioactive compounds and the lipid matrix, supporting successful nanoencapsulation

**Fig. 4:**
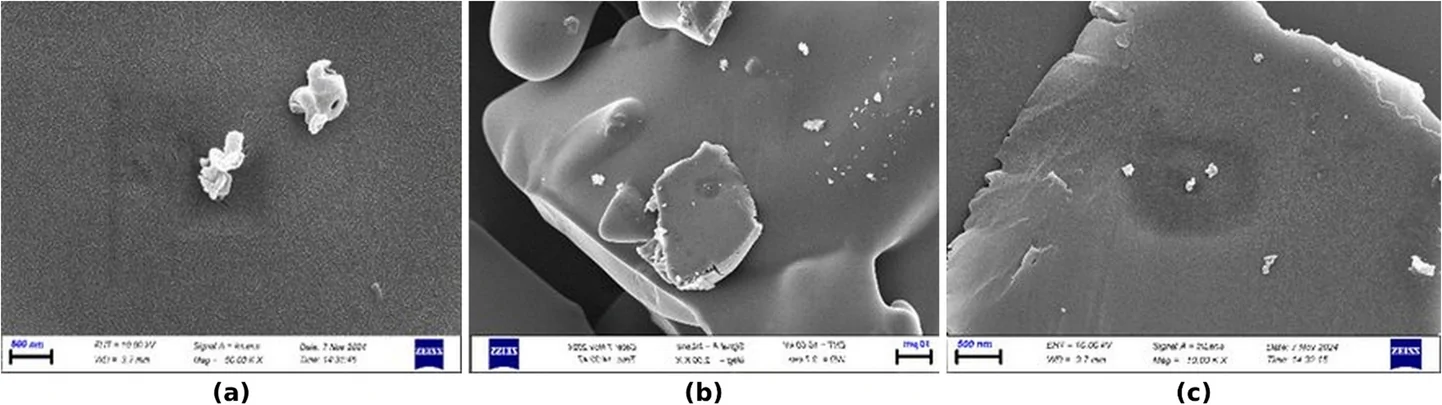
SEM micrographs of lyophilized *Artemisia haussknechtii* leaf ethanolic extract at different magnifications: (**a**) 50.00 K×, scale bar 500 nm; (**b**) 2.00 K×, scale bar 10 μm; (**c**) 10.00 K×, scale bar 500 nm. The free extract displays irregular, flake-like particle morphology with heterogeneous size distribution

**Fig. 5:**
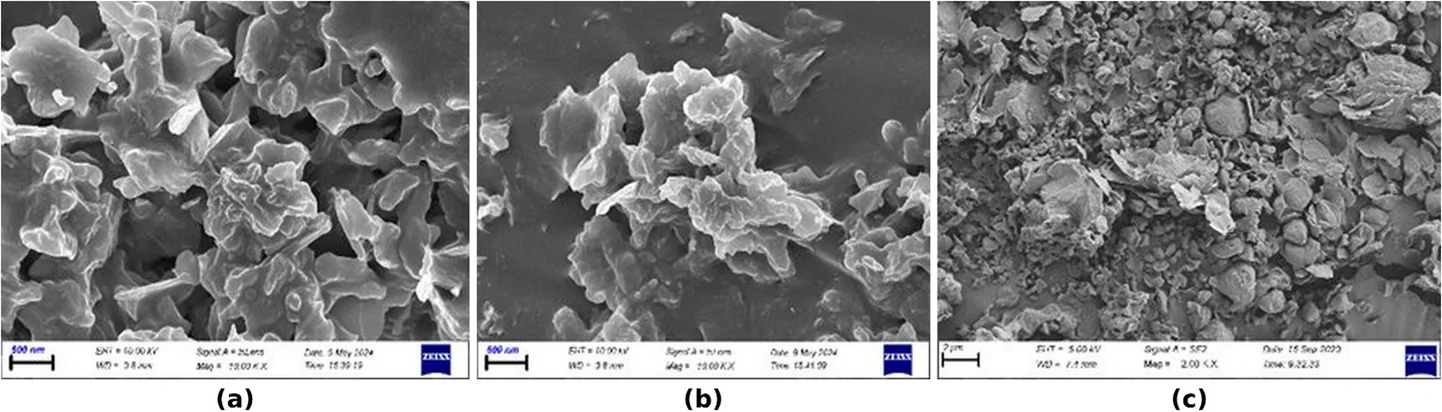
SEM micrographs of solid lipid nanoparticle-loaded *Artemisia haussknechtii* ethanolic extract at different magnifications: (**a**) 10.00 K×, scale bar 500 nm; (**b**) 10.00 K×, scale bar 500 nm; (**c**) 2.00 K×, scale bar 2 μm. The SLN formulation displays a more regular and homogeneous particle distribution compared to the free extract, consistent with successful nanoencapsulation

The physicochemical properties of the *Artemisia haussknechtii* leaf extract-loaded solid lipid nanoparticles (SLNs) were evaluated prior to animal administration. Zeta potential measurements of the free lyophilized ethanolic extract yielded an average value of 2.08 ± 12.75 mV, with a wide distribution among measurements indicating non-homogeneous surface charge. In contrast, the zeta potential of the SLN formulation was determined to be − 8.42 ± 2.45 mV, with measurements distributed within a narrower range, reflecting a more stable colloidal structure (Table 1). According to particle size analysis, the average hydrodynamic diameter of the SLN formulation was 173.1 ± 16.4 nm, and the polydispersity index (PDI) was calculated as 0.264 ± 0.036, indicating a relatively homogeneous size distribution. The LD10, LD50, and LD90 values were determined as 65.5 ± 2.0 nm, 145.7 ± 5.9 nm, and 478.6 ± 175.8 nm, respectively (Table 2). Particle size measurement could not be performed for the free ethanolic extract due to its physicochemical nature. FT-IR spectroscopic analysis of the lyophilized *A. haussknechtii* ethanolic extract revealed characteristic absorption bands at 3277.76 cm⁻¹ (O-H stretching, indicative of phenolic and hydroxyl groups), 2925.54 cm⁻¹ (C-H stretching of aliphatic chains), 1511.87 cm⁻¹ (C = O stretching), 1597.30 cm⁻¹ (C = C aromatic stretching), 1372.24 and 1262.84 cm⁻¹ (C-H and C-O bending/stretching), and 1045.23 cm⁻¹ (C-O-C stretching) (Fig. 2). In the SLN formulation spectrum, the characteristic bands of the plant extract were retained, with notable shifts observed: O-H stretching shifted to 3303.08 cm⁻¹, and a prominent band at 1734.34 cm⁻¹ corresponding to C = O ester stretching of the lipid matrix (Compritol) appeared, confirming successful integration of the extract within the lipid carrier. Additional bands at 2847.61 and 2914.20 cm⁻¹ (C-H stretching), 1601.73 cm⁻¹ (C = C aromatic), 1206.42 and 1071.53 cm⁻¹ (C-O-C stretching) were also identified (Fig. 3). The observed peak shifts between the free extract and SLN formulation spectra suggest physicochemical interaction between the bioactive compounds and the lipid matrix, supporting successful nanoencapsulation. Scanning electron microscopy (SEM) examination demonstrated that the free lyophilized extract exhibited an irregular, flake-like particle morphology with heterogeneous size distribution (Fig. 4), whereas the SLN formulation displayed a more regular and homogeneous particle distribution, consistent with the formation of a well-defined nanoparticulate system (Fig. 5). Thermogravimetric analysis (TGA) revealed that the mass loss profile of the SLN formulation differed from the thermal behavior of the free extract, indicating improved thermal stability of the bioactive compounds upon encapsulation within the lipid matrix.

**Table 1:** Zeta potential measurements of *Artemisia haussknechtii* leaf extract and solid lipid nanoparticle (SLN) formulation

Parameter	Lyophilized Ethanolic Extract	SLN Formulation
Zeta Potential (mV)	2.08 ± 12.75	−8.42 ± 2.45
Surface charge distribution	Wide (non-homogeneous)	Narrow (homogeneous)

**Table 2:** Particle size distribution and polydispersity index (PDI) of *Artemisia haussknechtii* extract-loaded solid lipid nanoparticles

Parameter	Value (Mean ± SD)
Average hydrodynamic diameter (nm)	173.1 ± 16.4
Polydispersity index (PDI)	0.264 ± 0.036
LD10 (nm)	65.5 ± 2.0
LD50 (nm)	145.7 ± 5.9
LD90 (nm)	478.6 ± 175.8

### Changes in Body Weight

As shown in Table 3 body weight significantly decreased in the diabetic control (DC) group at the end of the experimental period compared with the normal control (NC) group (*p* < 0.05). In contrast, diabetic rats treated with *Artemisia haussknechtii* extracts exhibited a partial prevention of body weight loss. This effect was more pronounced in the high-dose treatment groups (DYEE2 and DYNE2), with the DYNE2 group showing body weight values closest to those of the NC group.

**Table 3:** Body weight changes in rats treated with *Artemisia haussknechtii* Boiss. extracts during the experimental period

Groups Body Weight (g)	NC X ± SD	DC X ± SD	DYEE1 X ± SD	DYNE1 X ± SD	DYEE2 X ± SD	DYNE2 X ± SD
**Before treatment**	169.13 ± 4.28	176.28 ± 5.22	164.93 ± 9.11	159.86 ± 15.97	178.53 ± 4.87	176.02 ± 13.52
**After treatment**	184.37 ± 7.56	147.66 ± 4.90^a^	147.99 ± 6.96^ab^	154.81 ± 14.38^abc^	164.94 ± 9.89^abc^	171.30 ± 7.08^abcd^

ᵃ *p* < 0.05 vs. NC; ᵇ *p* < 0.05 vs. DC; ᶜ *p* < 0.05 vs. DYEE1; ᵈ *p* < 0.05 vs. DYNE1; ᵉ *p* < 0.05 vs. DYEE2.

### Fasting Blood Glucose Levels

Blood glucose levels measured throughout the experimental period are presented in Table 4. In the DC group, blood glucose levels progressively increased over time and remained significantly higher than those of the NC group at all measurement points (*p* < 0.05). In diabetic rats treated with *Artemisia haussknechtii* extracts, a time-dependent reduction in blood glucose levels was observed. Notably, significant decreases compared with the DC group were detected from Day 7 onward in the DYEE2 and DYNE2 groups (*p* < 0.05), with further reductions observed on Days 14 and 21. Among the treated groups, the DYNE2 group exhibited the lowest blood glucose levels during the experimental period.

**Table 4:** Changes in fasting blood glucose levels in rats treated with *Artemisia haussknechtii* Boiss. extracts during the experimental period

Blood Glucose (mg/dL)	NC X ± SD		DC X ± SD		DYEE1 X ± SD		DYNE1 X ± SD		DYEE2 X ± SD		DYNE2 X ± SD	
	0 h	3 h	0 h	3 h	0 h	3 h	0 h	3 h	0 h	3 h	0 h	3 h
**Day 0**	86.72 ± 10.08	86.82 ± 10.16	250.62± 26.85	258.65± 43.26	251.93± 36.13	236.47± 17.84	225.76± 12.97	219.55± 9.86	220.11± 6.25	216.29± 12.84	262.04± 19.20	244.04± 5.56
**Day 7**	86.66± 9.66	87.17± 9.50	354.37± 21.83^a^	354.37± 21.83	242.24± 9.80	237.85± 14.00	218.27± 9.35	217.47± 9.10	155.22± 10.88	154.31± 10.93	157.12± 6.72^b^	156.49± 6.73
**Day 14**	88.11± 8.96	88.42± 9.19	368.51± 13.90^a^	369.22± 13.69	223.41± 7.28	222.55± 7.29	214.18± 5.83	213.65± 5.80	137.03± 5.07^b^	136.82± 4.77	123.98± 5.65^b^	123.26± 5.65
**Day 21**	88.07± 9.34	88.01± 8.74	374.84± 12.66^a^	375.64± 13.09	205.59± 7.35	203.72± 9.09	197.13± 3.57	196.33± 3.70	108.37± 5.53^b^	107.27± 5.93	101.61± 7.00^b^	99.11 ± 5.58

ᵃ *p* < 0.05 vs. NC; ᵇ *p* < 0.05 vs. DC; ᶜ *p* < 0.05 vs. DYEE1; ᵈ *p* < 0.05 vs. DYNE1; ᵉ *p* < 0.05 vs. DYEE2

### HbA1c Levels

As shown in Table 5, serum insulin and C-peptide levels were significantly decreased, whereas HbA1c levels were significantly increased in the DC group compared with the NC group (*p* < 0.05). Administration of *Artemisia haussknechtii* extracts resulted in a reduction in HbA1c levels, particularly in the DYEE2 and DYNE2 groups, where values approached those of the NC group. Concurrently, insulin and C-peptide levels were significantly higher in these groups compared with the DC group.

**Table 5:** Serum insulin, HbA1c (%), and C-peptide levels in rats treated with *Artemisia haussknechtii* extract

Groups Parameters	NC	DC	DYEE1	DYNE1	DYEE2	DYNE2
	X ± SD	X ± SD	X ± SD	X ± SD	X ± SD	X ± SD
Insulin (pg/mL)	577.52 ± 47.37	412.7 ± 18.83^a^	450.06 ± 53.53^ab^	477.46 ± 21.08^ab^	556.68 ± 41.86^abc^	577.92 ± 22.78^abd^
HbA1c (%)	4.38 ± 0.44	7.12 ± 0.86^a^	6.38 ± 1.01^ab^	6.08 ± 0.61^ab^	4.44 ± 0.78^abc^	4.34 ± 0.20^ab^
C-Peptide (pg/mL)	572.04 ± 50.38	460.2 ± 27.22^a^	489.82 ± 66.89^ab^	486.88 ± 38.84^a^	534.2 ± 30.74^abc^	551.96 ± 13.67^ad^

ᵃ *p* < 0.05 vs. NC; ᵇ *p* < 0.05 vs. DC; ᶜ *p* < 0.05 vs. DYEE1; ᵈ *p* < 0.05 vs. DYNE1; ᵉ *p* < 0.05 vs. DYEE2

### Serum Biochemical Parameters

As summarized in Table 6, serum ALT, AST, and LDH activities were significantly elevated in the DC group compared with the NC group (*p* < 0.05). Treatment with *Artemisia haussknechtii* extracts resulted in a reduction of these enzyme activities, particularly in the high-dose groups. ALT, AST, and LDH levels in the DYEE2 and DYNE2 groups were significantly lower than those in the DC group, with the DYNE2 group showing values closer to normal control levels.

Regarding renal function parameters, serum urea and creatinine levels were significantly increased in the DC group relative to the NC group (*p* < 0.05). Administration of *Artemisia haussknechtii* extracts led to a marked decrease in these parameters, with the most pronounced reductions observed in the DYEE2 and DYNE2 groups.

**Table 6:** Serum liver and kidney function parameters in diabetic rats treated with *Artemisia haussknechtii* extract

Groups Parameters	NC X ± SD	DC X ± SD	DYEE1 X ± SD	DYNE1 X ± SD	DYEE2 X ± SD	DYNE2 X ± SD
ALT U/L	72.65 ± 19.75	177.75 ± 25.53^a^	168.89 ± 7.63^a^	160.84 ± 9.33^a^	135.96 ± 6.77^ac^	124.91 ± 10.82^ae^
AST U/L	148.19 ± 23.08	243.89 ± 27.96^a^	217.85 ± 114.81^a^	213.06 ± 5.22^a^	182.45 ± 14.75^abc^	158.32±10.48^abde^
Urea mg/dL	49.15 ± 3.51	87.51 ± 1.19^a^	83.27 ± 1.77^ab^	81.79 ± 2.54^ab^	65.03 ± 2.20^abc^	60.19 ± 0.84^abe^
Creatinine mg/dL	36.00 ± 0.87	53.40 ± 1.08^a^	51.39 ± 1.81^ab^	49.06 ± 1.10^ab^	42.12 ± 1.08^abc^	40.19 ± 1.49^abd^
LDH U/L	1611.19 ± 22.88	2124.34 ± 40.97^a^	2067.65 ± 21.04^ab^	2014.83 ± 28.77^ab^	1778.27 ± 34.89^abc^	1741.91 ± 32.62^abe^

ᵃ *p* < 0.05 vs. NC; ᵇ *p* < 0.05 vs. DC; ᶜ *p* < 0.05 vs. DYEE1; ᵈ *p* < 0.05 vs. DYNE1; ᵉ *p* < 0.05 vs. DYEE2

Lipid profile parameters are presented in Table 7. The DC group showed significantly elevated levels of total cholesterol, triglycerides, and LDL-cholesterol, accompanied by a significant reduction in HDL-cholesterol compared with the NC group (*p* < 0.05). Treatment with *Artemisia haussknechtii* extracts partially ameliorated these alterations. HDL-cholesterol levels increased toward normal values, while total cholesterol levels were notably reduced in the treated groups. The most pronounced improvement in lipid profile parameters was observed in the DYNE2 group.

**Table 7:** Serum lipid profile in rats treated with *Artemisia haussknechtii* extract

Groups Paramaters	NC X ± SD	DC X ± SD	DYEE1 X ± SD	DYNE1 X ± SD	DYEE2 X ± SD	DYNE2 X ± SD
HDL (mg/dL)	48.06 ± 0.71	35.4 ± 0.60^a^	47.3 ± 1.43^ab^	47.64 ± 1.04^ab^	46.58 ± 1.06^abc^	46.2 ± 1.18^abe^
LDL (mg/dL)	8.22 ± 0.28	12.4 ± 0.44^a^	10.96 ± 0.46^ab^	11.68 ± 0.54^ab^	12.38 ± 0.88^abc^	10.6 ± 0.93^abe^
Triglycerides (mg/dL)	31.14 ± 14.74	45.52 ± 7.25^a^	37.4 ± 6.83^a^	44.54 ± 8.66^a^	36.14 ± 7.57^a^	34.54 ± 11.93^a^
Total Cholesterol (mg/dL)	32.84 ± 6.66	68.22 ± 11.20^a^	49.1 ± 3.63^ab^	46.16 ± 7.20^ab^	38.9 ± 3.11^abc^	36.56 ± 5.01^abe^

ᵃ *p* < 0.05 vs. NC; ᵇ *p* < 0.05 vs. DC; ᶜ *p* < 0.05 vs. DYEE1; ᵈ *p* < 0.05 vs. DYNE1; ᵉ *p* < 0.05 vs. DYEE2

### Changes in Oxidative Stress and Antioxidant Parameters

Oxidative stress and antioxidant parameters measured in erythrocyte, liver, and kidney tissues are summarized in Table 8. In the DC group, malondialdehyde (MDA) and total oxidant status (TOS) levels were significantly increased across all tissues, while antioxidant parameters including GSH, GPx, GR, CAT, and TAS were significantly decreased compared with the NC group (*p* < 0.05).

Treatment with *Artemisia haussknechtii* extracts led to a reduction in oxidative stress markers and an enhancement of antioxidant defense systems in a dose-dependent manner. These effects were most pronounced in the DYNE2 group, where MDA and TOS levels were markedly reduced and antioxidant parameters were restored toward normal control values.

**Table 8:** Lipid peroxidation, antioxidant defense systems, and TAS/TOS levels in rats treated with *Artemisia haussknechtii* extract

Tissues	Groups Parameters	NC X ± SD	DC X ± SD	DYEE1 X ± SD	DYNE1 X ± SD	DYEE2 X ± SD	DYNE2 X ± SD
**Erythrocyte (U/g)**	MDA	9.44 ± 1.41	13.40 ± 0.79^a^	9.94 ± 0.97^ab^	10.26 ± 0.98^abc^	9.33 ± 1.09^abc^	8.31 ± 1.30^abe^
	GSH	5.62 ± 0.29	3.84 ± 0.17^a^	4.04 ± 0.48^ab^	4.33 ± 0.47^abc^	4.71 ± 0.32^abc^	5.32 ± 0.60^abde^
	GST	9.09 ± 1.03	14.21 ± 0.84^a^	14.18 ± 1.21^ab^	12.97 ± 1.22^abc^	111.68 ± 1.30^abc^	10.45 ± 0.72^abde^
	GR	5.15 ± 0.04	3.11 ± 0.02^a^	3.19 ± 0.01^ab^	3.30 ± 0.01^abc^	4.05 ± 0.01^ab^	4.38 ± 0.01^abde^
	CAT	371.86 ± 28.16	483.03 ± 23.45^a^	466.25 ± 26.55^a^	451.07 ± 30.25^a^	418.48 ± 28.23^abc^	394.12 ± 37.02^abd^
	GPX	128.31 ± 6.30	95.24 ± 2.44^a^	96.98 ± 5.91^ab^	105.92 ± 6.85^abc^	120.95 ± 3.82^abc^	123.74 ± 4.07^abde^
	SOD	152.63 ± 7.13	176.64 ± 7.45^a^	171.96 ± 10.53^ab^	169.62 ± 6.24^ab^	160.88 ± 3.95^abc^	159.47 ± 8.76^ab^
	TAS	2.23 ± 0.29	1.45 ± 0.13^a^	1.65 ± 0.07^ab^	1.68 ± 0.03^abc^	1.87 ± 0.09^abc^	1.88 ± 0.10^abde^
	TOS	3.82 ± 0.04	8.68 ± 0.16^a^	8.09 ± 0.12	7.57 ± 0.40	5.80 ± 0.86	4.72 ± 0.26^b^
**Liver (U/g)**	MDA	167.14 ± 13.06	233.11 ± 8.58^a^	213.70 ± 6.68^ab^	207.06 ± 9.63^abc^	181.25 ± 9.16^abc^	178.43 ± 11.45^abde^
	GSH	50.69 ± 3.15	41.27 ± 2.59^a^	43.59 ± 1.95^ab^	45.53 ± 1.02^abc^	48.62 ± 2.00^abc^	49.54 ± 1.10^abde^
	GST	62.48 ± 6.22	78.79 ± 4.44^a^	74.56 ± 3.52^ab^	71.47 ± 5.20^abc^	65.1 ± 5.23^abc^	63.61 ± 1.49^abde^
	GR	0.47 ± 0.06	0.36 ± 0.05^a^	0.36 ± 0.05^ab^	0.37 ± 0.06^abc^	0.43 ± 0.03^abc^	0.44 ± 0.02^abde^
	CAT	446.86 ± 9.33	319.21 ± 14.23^a^	320.34 ± 22.99^ab^	350.58 ± 17.03^abc^	414.12 ± 17.25^ab^	420.59 ± 11.68^abd^
	GPX	72.79 ± 6.73	54.55 ± 8.73^a^	55.31 ± 1.38^ab^	59.77 ± 4.18^abc^	67.35 ± 3.04^abc^	67.57 ± 5.91^abde^
	SOD	107.02 ± 3.12	132.62 ± 3.13^a^	131.54 ± 4.01^ab^	128.26 ± 4.56^bc^	125.02 ± 2.24^b^	122.25 ± 1.97^be^
	TAS	3.02 ± 0.10	1.62 ± 0.08^a^	1.83 ± 0.12	2.17 ± 0.18	2.56 ± 0.07	2.71 ± 0.07^b^
	TOS	3.84 ± 0.26	10.80 ± 0.17^a^	10.37 ± 0.12	10.21 ± 0.09	8.00 ± 0.05	6.56 ± 0.16^b^
**Kidney (U/g)**	MDA	53.68 ± 6.61	69.88 ± 2.94^a^	64.56 ± 3.24^ab^	60.25 ± 4.06^ab^	59.24 ± 4.09^abc^	56.71 ± 2.28^abd^
	GSH	59.94 ± 1.34	43.72 ± 1.41^a^	45.48 ± 7.02^ab^	46.06 ± 5.11^abc^	51.83 ± 2.81^abc^	57.84 ± 3.69^abde^
	GST	8.35 ± 0.66	14.66 ± 0.82^a^	14.14 ± 1.70^ab^	13.01 ± 1.05^abc^	11.53 ± 1.20^abc^	10.35 ± 1.57^abde^
	GR	0.79 ± 0.11	0.46 ± 0.08^a^	0.52 ± 0.06^ab^	0.56 ± 0.05^abc^	0.71 ± 0.04^abc^	0.77 ± 0.09^abde^
	CAT	320.83 ± 10.00	207.00 ± 7.4^a^	237.02 ± 10.54^ab^	242.99 ± 13.07^abc^	277.07 ± 6.40^abc^	296.21 ± 9.67^abde^
	GPX	82.66 ± 5.25	62.07 ± 4.18^a^	63.75 ± 2.49^ab^	67.45 ± 5.98^abc^	76.33 ± 3.66^abc^	8.82 ± 4.91^abde^
	SOD	102.62 ± 4.05	83.94 ± 3.28^a^	88.50 ± 3.67^ab^	88.55 ± 3.90^abc^	97.56 ± 2.54^ac^	98.45 ± 4.23^ade^
	TAS	1.88 ± 0.20	0.54 ± 0.17^a^	0.71 ± 0.10	0.93 ± 0.06	1.39 ± 0.09^bc^	1.60 ± 0.03^bd^
	TOS	3.97 ± 0.16	10.17 ± 0.21^a^	9.35 ± 0.39	9.09 ± 0.43	6.93 ± 0.16	6.52 ± 0.04^b^

ᵃ *p* < 0.05 vs. NC; ᵇ *p* < 0.05 vs. DC; ᶜ *p* < 0.05 vs. DYEE1; ᵈ *p* < 0.05 vs. DYNE1; ᵉ *p* < 0.05 vs. DYEE2

### Histopathological Findings

**Fig. 6:**
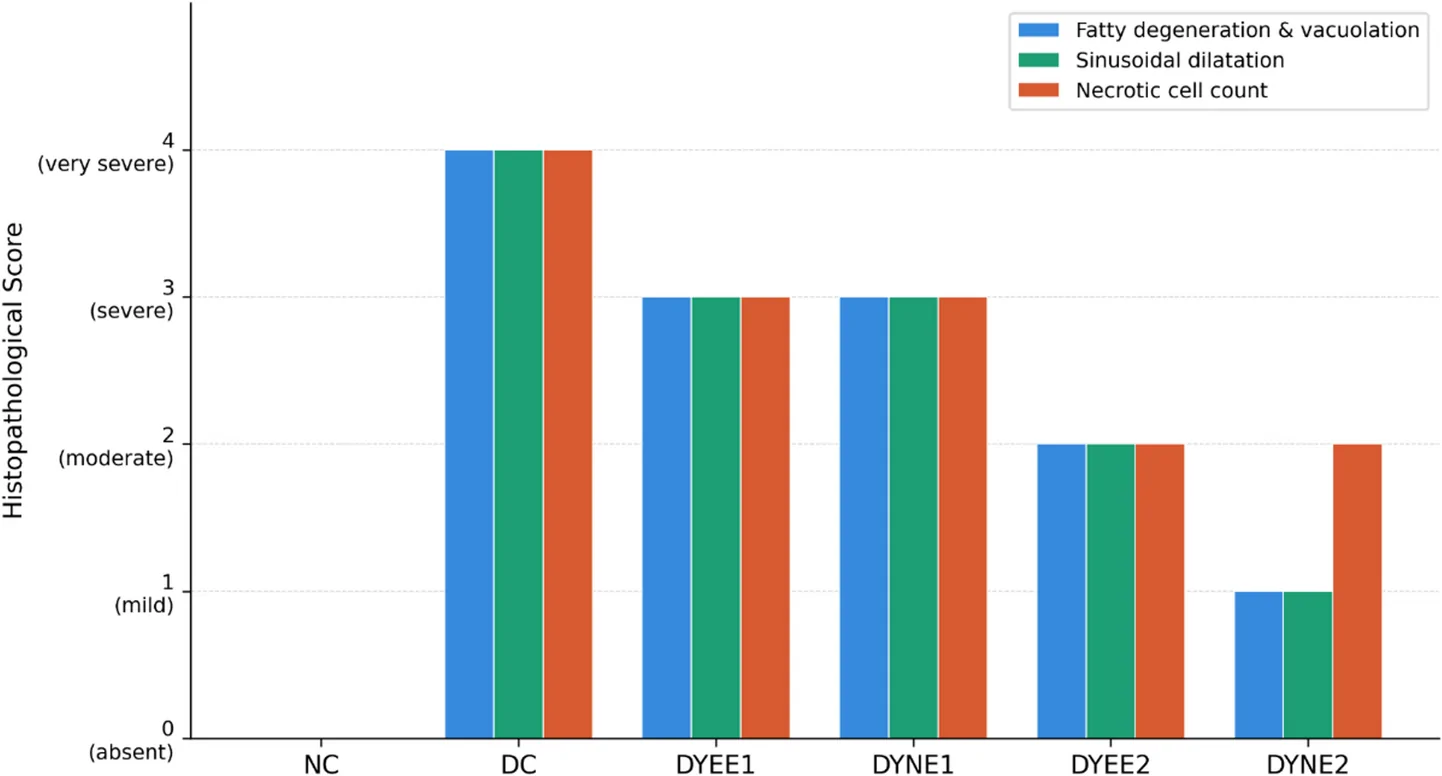
Histopathological scores of liver tissue

**Fig. 7:**
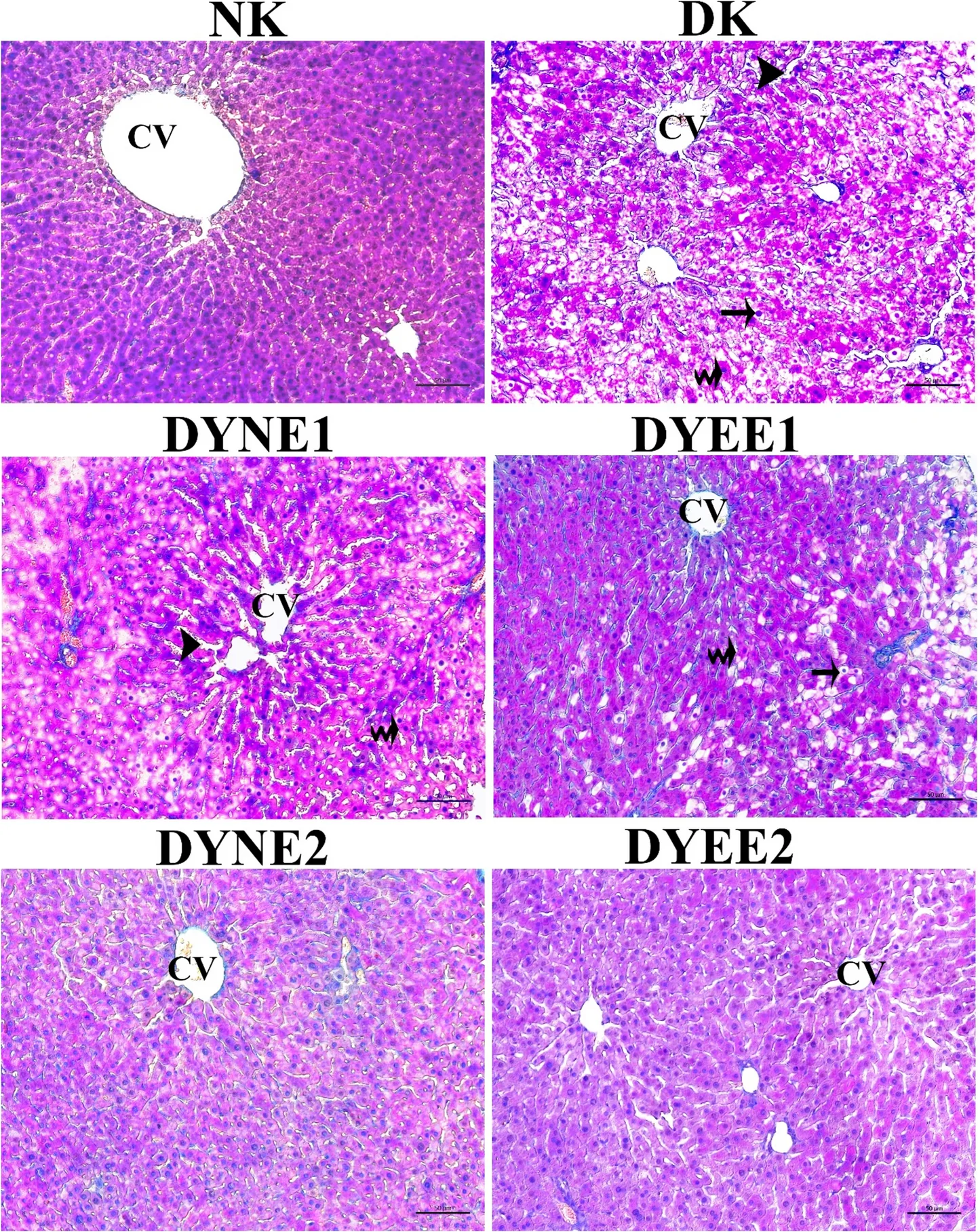
Photomicrographs of liver tissues from control and experimental groups (Masson’s trichrome staining; objective lens: ×20; scale bar: 50 μm). CV: central vein; arrow: fatty degeneration and vacuolization; arrowhead: sinusoidal dilatation; curved arrow: necrotic cells

Histopathological examinations revealed that the diabetic group exhibited pronounced structural alterations in the liver, kidney, and pancreatic tissues. Histopathological examination of liver tissue revealed normal hepatic architecture in the control group (Fig. 6). In the diabetic group, severe fatty degeneration and vacuolization, marked sinusoidal dilatation, and increased necrotic cell numbers were evident (Fig. 7). These alterations were partially reduced in the DYNE1 and DYEE1 groups. In contrast, the DYNE2 group showed marked histological improvement, characterized by minimal fatty vacuolization and near-normal sinusoidal morphology, while the DYEE2 group exhibited comparable findings (Fig. 6). Histopathological scores for all groups are presented in Fig. 7.

**Fig. 8:**
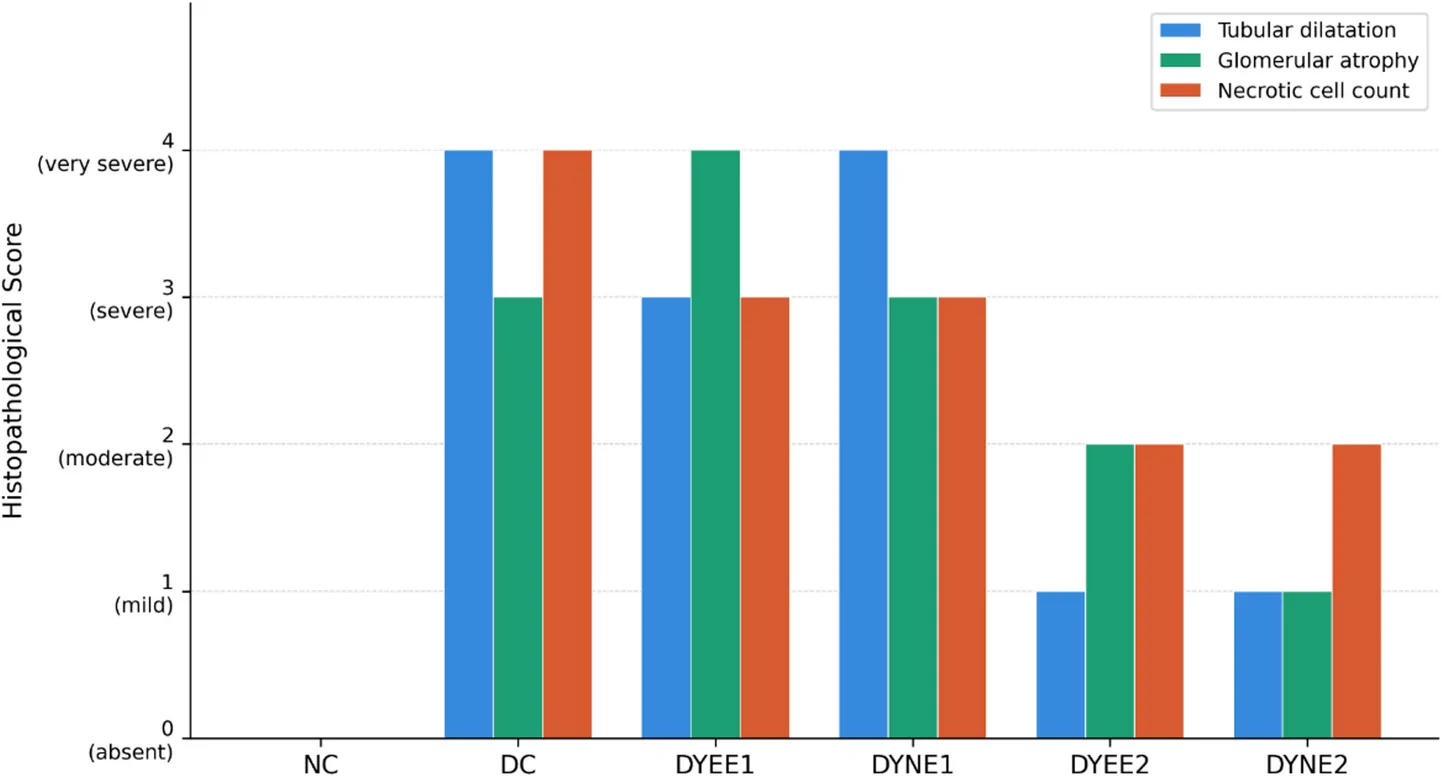
Histopathological scores of kidney tissue

**Fig. 9:**
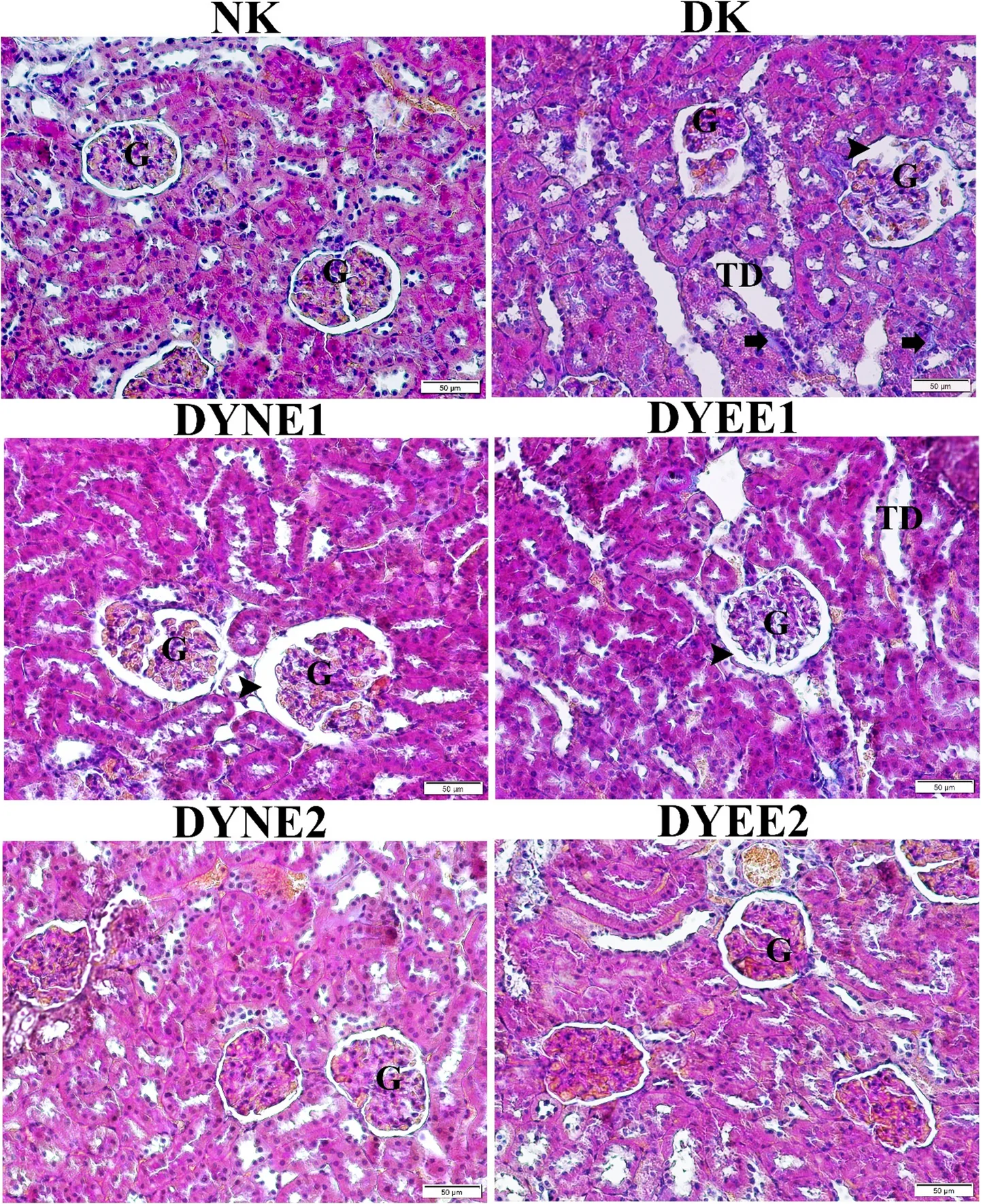
Photomicrographs of kidney tissue from the control and experimental groups (Masson’s trichrome staining; objective lens: ×20; scale bar: 50 µm). G: glomerulus; arrowhead: separated Bowman’s capsule; thick arrow: collagen accumulation; TD: tubular dilatation

Histopathological examination of kidney tissue revealed normal renal architecture in the control group, with intact glomeruli and preserved tubular morphology (Fig. 8). In the diabetic group, pronounced renal damage was observed, characterized by severe tubular dilatation, hydropic degeneration and necrosis of tubular epithelial cells, and glomerular atrophy. These alterations were partially reduced in the DYNE1 and DYEE1 groups. In contrast, the DYNE2 group demonstrated marked histological improvement, with better preservation of glomerular structure and tubular organization. Similar findings were observed in the DYEE2 group (Fig. 8). Histopathological scores for all groups are presented in Fig. 9.

**Fig. 10:**
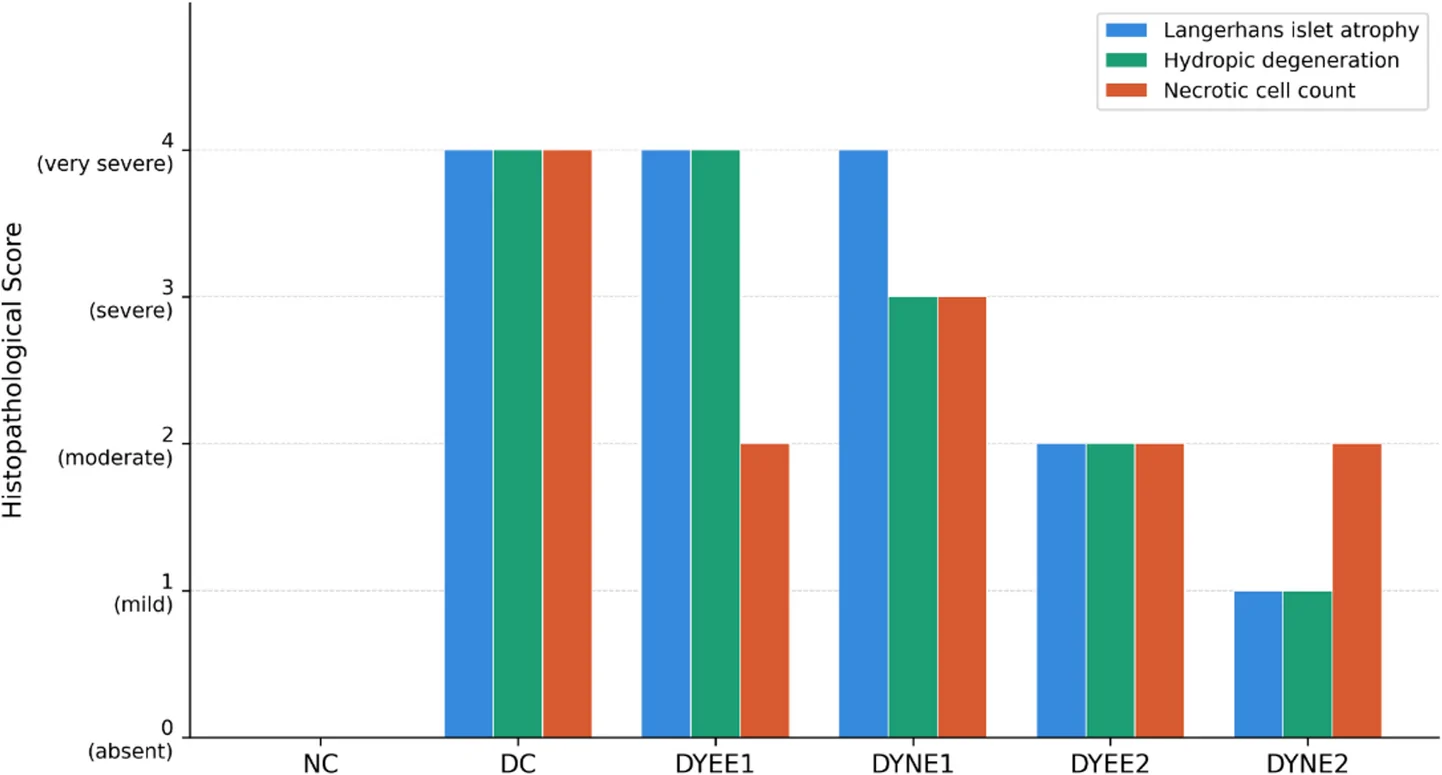
Histopathological scores of pancreatic tissue

**Fig. 11:**
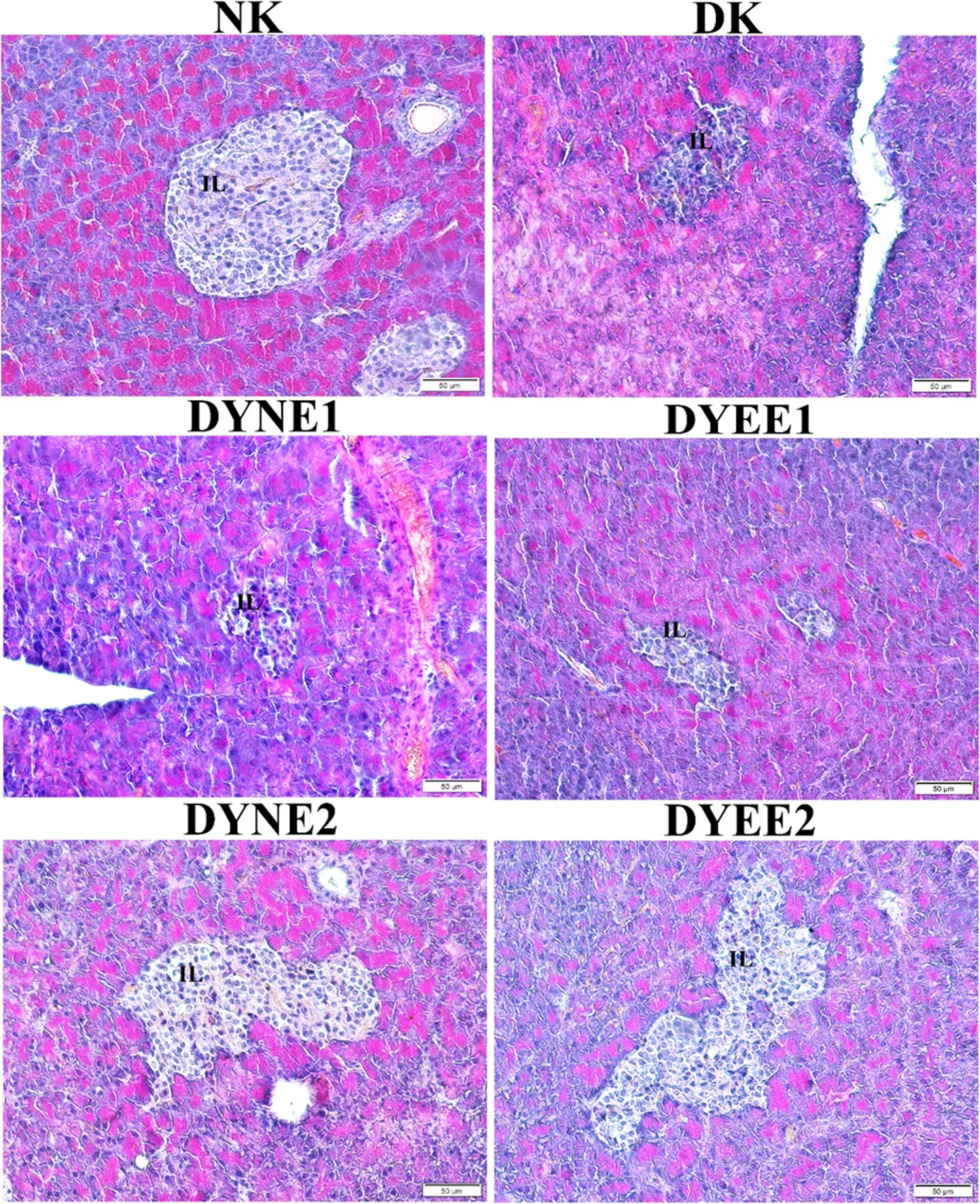
Photomicrographs of pancreatic tissue from the control and experimental groups (Masson’s trichrome staining; objective lens: ×20; scale bar: 50 µm). IL: islets of Langerhans

Histopathological examination of pancreatic tissue revealed normal Langerhans islet architecture in the control group. In contrast, the diabetic group showed severe islet atrophy, marked cellular loss, hydropic degeneration, and increased necrotic cells (Fig. 10). The DYNE1 and DYEE1 groups exhibited limited improvement, whereas the DYNE2 and DYEE2 groups showed clearer preservation of islet structure and reduced degenerative and necrotic changes compared to the diabetic group, consistent with the histopathological scores (Fig. 11).

## Discussion

Diabetes mellitus (DM) is a metabolic disease and is recognized as a growing public health problem worldwide. In this study, the ethanol extract of *Artemisia haussknechtii* Boiss. and the nanoparticle form of the extract were evaluated in rats with STZ-induced diabetes in terms of biochemical, oxidative stress, and histopathological parameters. The findings indicate that diabetes causes serious impairments in metabolic and cellular processes and that herbal treatment approaches can partially correct these impairments.

Hyperglycemia, one of the key indicators of diabetes, has increased significantly in the diabetic control group. The dramatic rise in blood glucose levels is due to the toxic effect of STZ on pancreatic β-cells and is consistent with similar models reported in the literature [61, 62]. Significant decreases in glucose levels were observed in the groups treated with *Artemisia haussknechtii* extract, particularly in the DYEE2 and DYNE2 groups. This indicates that the extract produces a more pronounced antidiabetic effect at high doses and in nanoparticle form. The antihyperglycemic effects observed may be attributed to the inhibition of α-glucosidase and α-amylase enzymes by phenolic compounds present in *Artemisia haussknechtii*, thereby reducing intestinal glucose absorption. Additionally, flavonoids such as quercetin and luteolin reported in Artemisia species have been shown to stimulate insulin secretion from residual β-cells and enhance peripheral glucose uptake via activation of the PI3K/Akt insulin signaling pathway [63, 65]. Previous studies have indicated that plant-based antioxidant components may alleviate impaired glucose metabolism [63]. Similarly, Helal et al. [64] reported the hypoglycemic and insulin-sensitizing effects of Artemisia annua extract in alloxan-induced diabetic models. Furthermore, Sharifi-Rad et al. [65] systematically demonstrated that Artemisia species have antioxidant, anti-inflammatory, and metabolic disease-improving potential, but emphasized that clinical and toxicological data are limited.

Metabolic disorders associated with STZ-diabetes have led to weight loss. The weight loss recorded in the diabetic control group is a result of catabolic processes triggered by insulin deficiency [66]. In groups administered Artemisia extract, particularly in nanoparticle form, this loss was partially prevented. This supports the extract’s effect in alleviating metabolic imbalance and is consistent with other herbal studies reported in the literature [46, 67].

In the diabetic control group, the increase in MDA and TOS levels and the decrease in antioxidant defense parameters such as GSH, GST, GPx, SOD, CAT, and TAS indicate increased free radical production and elevated oxidative damage risk in diabetes [68, 69]. *Artemisia haussknechtii* extract, particularly in nanoparticle form, significantly corrected these impairments; it reduced MDA and TOS levels while increasing antioxidant enzyme activities. The restoration of antioxidant enzyme activities observed in extract-treated groups may be mechanistically linked to the activation of the Nrf2/ARE (Nuclear factor erythroid 2-related factor 2/Antioxidant Response Element) signaling pathway by polyphenolic constituents of *Artesemia haussknechtii*. Nrf2 activation upregulates the transcription of cytoprotective enzymes including SOD, CAT, GPx, and GR, thereby reinforcing the cellular antioxidant defense system against ROS-induced damage [70, 72]. These effects are consistent with the plant’s phenolic compound-rich structure and its capacity to eliminate reactive oxygen species [70–72]. Similarly, the literature reports that Artemisia species have a protective effect on diabetes-related oxidative stress and increase antioxidant enzyme activities [73]. The biochemical findings obtained in our study were also reflected at the histopathological level; degenerative changes observed in the liver, kidney, and pancreas were significantly reduced in high-dose and nanoparticle applications. These results support the therapeutic effects of Artemisia species on the pathophysiological processes of diabetes. It is known that reactive oxygen species, which increase due to hyperglycemia, trigger oxidative stress and inflammation, whereas antioxidant compounds can mitigate these effects by regulating glucose metabolism. Flavonoids have been reported to exhibit both metabolic regulatory and antioxidant activities on biological targets associated with diabetes [74]. This information scientifically supports the effects of *Artemisia haussknechtii* extract in reducing oxidative stress and metabolic disorders in our diabetic model.

Additionally, a significant improvement in lipid profile parameters was observed in the groups treated with Artemisia extract; the nanoparticle form specifically increased HDL levels while decreasing LDL and triglyceride levels. These results are consistent with previous studies reporting the antidiabetic and anti-obesity potential of Artemisia species [75, 76].

Histopathological examinations revealed degenerative changes in the liver, kidney, and pancreas tissues of the diabetic control group: fatty degeneration and sinusoidal dilatation in the liver; hydropic degeneration and glomerular damage in the kidney; and cell loss and structural deterioration in the islets of Langerhans in the pancreas. These changes were significantly reduced in the groups treated with the extract, and the morphological integrity of the tissues was preserved, particularly in the DYNE2 group. The attenuation of hepatic and renal tissue damage observed in treated groups may be related to the suppression of NF-κB-mediated inflammatory cascades by bioactive terpenoids and flavonoids in the extract. Inhibition of NF-κB reduces the production of pro-inflammatory cytokines such as TNF-α and IL-6, which are known mediators of diabetes-induced organ fibrosis and cellular necrosis [74, 75]. The superior efficacy of the nanoparticle formulation may be partly attributed to the physicochemical advantages conferred by the SLN system, such as nanometric particle size and lipid-based matrix, which have been suggested to improve cellular uptake and prolong retention in previous studies [44, 45, 77]; however, direct pharmacokinetic evidence was not obtained in the present study and warrants further investigation.

Similar studies have demonstrated that Artemisia species have beneficial effects on biochemical, oxidative, and histopathological parameters in diabetic models. Botrous et al. [80] reported that Artemisia annua extract reduced the side effects of pioglitazone treatment, while Albasher et al. [34] and Sekiou et al. [81] demonstrated that Artemisia judaica and *A. herba-alba* extracts protect liver and kidney tissues against diabetes-related damage. Furthermore, Mohamed et al. [46] reported that nanoformulations provide more pronounced metabolic and antioxidant benefits compared to conventional plant extracts. This literature supports the therapeutic effects of *Artemisia haussknechtii* obtained in our study, which are particularly pronounced with nanoparticle formulation. However, the present study differs from previous Artemisia research in several important aspects. First, while most existing studies have focused on widely investigated species such as *A. annua*, *A. judaica*, and *A. herba-alba*, the current study is among the first to evaluate the antidiabetic and tissue-protective potential of *Artemisia haussknechtii* Boiss., a species endemic to Eastern Anatolia with limited pharmacological documentation. Second, unlike previous studies that employed conventional plant extracts, this study simultaneously compared lyophilized ethanolic extract and solid lipid nanoparticle formulations at two dose levels, allowing a direct assessment of the impact of nanoencapsulation on therapeutic efficacy. Third, the comprehensive evaluation of oxidative stress parameters across three tissue compartments (erythrocyte, liver, and kidney) simultaneously, together with histopathological scoring of three organs (liver, kidney, and pancreas), provides a more integrative picture of diabetes-induced systemic damage and its attenuation compared to studies that assessed only selected parameters or single organ systems [34, 73, 80, 81]. When compared with similar nanoformulation studies in the literature, the findings of the present study are largely consistent. Mohamed et al. [46] reported that *A. annua* extract nanoparticles produced more pronounced improvements in glucose metabolism and oxidative stress markers compared to the conventional extract in hypercaloric diet-fed rats, which aligns with the superior efficacy of DYNE2 observed in our study. Similarly, Alshehri [47] demonstrated that *A. herba-alba* nanoparticles exhibited significant antioxidant and hypolipidemic effects in rats, comparable to the lipid profile improvements observed in our DYNE groups. Regarding oxidative stress parameters, the MDA reduction and antioxidant enzyme restoration observed in our study are consistent with those reported by Yazdi et al. [73] and Sekiou et al. [81], who demonstrated hepatoprotective and nephroprotective effects of *Artemisia* species in STZ-induced diabetic models. The histopathological improvements observed in liver, kidney, and pancreatic tissues in our high-dose nanoparticle group are also in agreement with findings reported by Albasher et al. [34] and Botrous et al. [80], further supporting the tissue-protective potential of *Artemisia*-based formulations in experimental diabetes.

The findings are consistent with previous studies reporting the positive effects of Artemisia species on diabetes, oxidative stress, and metabolic disorders. The literature indicates that Artemisia has therapeutic potential in various metabolic disorders such as liver toxicity, diabetes, and obesity [29, 30]. Similarly, toxicological evaluations conducted on Artemisia cina have shown that the plant components are safe at therapeutic doses and only induce limited oxidative stress at high concentrations [82]. This suggests that *Artemisia haussknechtii* exhibited a safe profile at appropriate doses in our study.

This study has demonstrated that *Artemisia haussknechtii* extract has the potential to alleviate metabolic disorders, reduce oxidative stress, and improve tissue damage in diabetic rats. In particular, the more pronounced therapeutic effect of the nanoparticle form indicates that increasing the bioavailability of plant components may enhance treatment efficacy. Reports in the literature on the antidiabetic, antioxidant, hepatoprotective, and cardioprotective effects of different Artemisia species [78, 79, 83, 84] support the scientific basis of the findings obtained in this study. Therefore, *Artemisia haussknechtii* is suitable for evaluation as a complementary phytotherapeutic agent against the pathophysiological processes of diabetes. However, comprehensive pharmacokinetic and toxicological studies are required to confirm its efficacy at the molecular level and clarify its safety profile.

## Conclusion

In conclusion, this study provides the first comprehensive evidence that ethanolic lyophilized extract and solid lipid nanoparticle formulation of *Artemisia haussknechtii* Boiss. exert significant antihyperglycemic, antioxidant, hypolipidemic, and tissue-protective effects in STZ-induced diabetic rats. The nanoparticle formulation consistently produced superior therapeutic outcomes compared to the free extract across all evaluated parameters, highlighting the critical role of nanotechnological delivery systems in potentially increasing the bioavailability and efficacy of plant-derived bioactive compounds. The main contribution of this study lies in being among the first to document the antidiabetic potential of *Artemisia haussknechtii* at both biochemical and histopathological levels, and in demonstrating that SLN-based nanoencapsulation substantially amplifies these effects through improved cellular delivery and sustained release mechanisms. These findings position *Artemisia haussknechtii* as a promising candidate for complementary phytotherapy in diabetes management. Future studies should focus on identifying and isolating the specific bioactive compounds responsible for the observed effects, elucidating their molecular targets and signaling pathways, conducting comprehensive pharmacokinetic and long-term toxicological evaluations, and ultimately designing controlled clinical trials to assess the translational potential of this plant extract in human subjects.

## Data Availability

The data that support the findings of this study are available from the corresponding author upon reasonable request.
